# The Use of Tooth Brushes

**Published:** 1883-10

**Authors:** 


### THE USE OF TOOTH BRUSHES.

If dealers will but call the attention of customers to the following rules for the use of brushes, so much complaint will not be made about their wearing qualities:

Directions for using tooth brushes—Tap the brush before using it, to see if you can jerk out any loose bristles.

Tap the brush after using it, to shake out the water, and put it away fairly dry.

Do not keep it closely shut up in a brush tray or dressing-bag bottle.

Causes of complaint—Loose bristles may be found in a new brush in consequence of the wire having cut the bristles in half while drawing them into the hole, the knot being too full.

Bristles may project beyond the level of the rest, the knot being too slack; clip them off; do not withdraw them and thereby make the knot still more slack.

Bristles will perish if brushes are put away while still wet, and left for days to get thoroughly dry; after a time even with the greatest care this will happen.

Brushes will smell offensively if closely shut up when wet; they will also become discolored.

Tooth brushes will wear out in course of time; some people use them for months, while some will cut them down very quickly, thus:—

Teeth with sharp edges will cut bristles.

Teeth with irregular spaces will catch individual bristles and forcibly withdraw them.

Some people select a brush too soft for their requirements, and make it harder by pressure, breaking down the bristles, which they would not do if their brush was sufficiently hard.

A tooth brush being an inexpensive article, it is wiser, therefore, to throw it aside before it is thoroughly worn out, than to keep it as an annoyance, which it will be if used too long.

